# Mapping suitable habitat for Nigeria–Cameroon chimpanzees in Kom-Wum Forest Reserve, North-Western Cameroon

**DOI:** 10.1007/s10329-023-01054-z

**Published:** 2023-02-18

**Authors:** Chefor Fotang, Udo Bröring, Christian Roos, Paul Dutton, Luc Roscelin Dongmo Tédonzong, Jacob Willie, Tsi Evaristus Angwafo, Yisa Ginath Yuh, Peter Schierack, Klaus Birkhofer

**Affiliations:** 1 Department of Ecology, Brandenburg University of Technology Cottbus-Senftenberg, Konrad-Wachsmann-Allee 6, 03046 Cottbus, Germany; 2 German Primate Center, Gene Bank of Primates and Primate Genetics Laboratory, Leibniz Institute for Primate Research, Göttingen, Germany; 3 Freelance Researcher, Waikato, New Zealand; 4 Centre for Research and Conservation (CRC), Royal Zoological Society of Antwerp (RZSA), Antwerp, Belgium; 5 Terrestrial Ecology Unit (TEREC), Department of Biology, Ghent University (UGent), Ghent, Belgium; 6 Faculty of Agronomy and Agricultural Sciences (FASA), University of Dschang, Dschang, Cameroon; 7 Hochschule fur nachhaltige Entwicklung Eberswalde, Forestry and Environment, Schicklerstraße 5, 16225 Eberswalde, Germany; 8 Szkola Glowna Gospodarstwa Wiejskiego, Nowoursynowska 166, 02-787 Warsaw, Poland; 9 Concordia University, Montreal, QC Canada; 10 Faculty of Environment and Natural Sciences, Brandenburg University of Technology Cottbus-Senftenberg, Senftenberg, Germany

**Keywords:** Environmental factors, Habitat requirements, Human impact, Protected area, Maximum entropy, *Pan troglodytes ellioti*

## Abstract

Great apes lose suitable habitats required for their reproduction and survival due to human activities across their distribution range in Africa. Little is known about habitat suitability of the Nigeria–Cameroon chimpanzee [*Pan troglodytes ellioti* (Matschie, 1914)], particularly for populations inhabiting forest reserves in North-West Cameroon. To address this knowledge gap, we employed a common species distribution model (MaxEnt) to map and predict suitable habitats for the Nigeria–Cameroon chimpanzee in Kom-Wum Forest Reserve, North-West Cameroon, based on environmental factors that potentially affect habitat suitability. We related these environmental factors to a dataset of chimpanzee occurrence points recorded during line transect and reconnaissance (recce) surveys in the forest reserve and surrounding forests.  Up to 91% of the study area is unsuitable for chimpanzees. Suitable habitats only represented 9% of the study area, with a high proportion of highly suitable habitats located outside the forest reserve. Elevation, secondary forests density, distance to villages and primary forests density were the most important predictors of habitat suitability for the Nigeria–Cameroon chimpanzee. The probability of chimpanzee occurrence increased with elevation, secondary forest density and distance from villages and roads. Our study provides evidence that suitable chimpanzee habitat in the reserve is degraded, suggesting that efforts to maintain protected areas are insufficient. The reserve management plan needs to be improved to conserve the remaining suitable habitat and to avoid local extinction of this endangered subspecies.

## Introduction

Human activities such as deforestation and forest degradation are causing continuous declines in the habitat suitability for terrestrial mammals worldwide (Pereira et al. 2010; Hansen et al. 2013; Newbold et al. 2015). Junker et al. (2012) reported a dramatic decline in the amount of suitable habitat for great apes across Africa over the last two decades. This decline is due to the conversion of forest to farmlands, commercial logging, industrial agriculture and road infrastructure development (Kormos et al. 2003; Zimmerman and Kormos 2012; Morgan et al. 2013; Laurance et al. 2014; Rainer et al. 2015). The availability of suitable habitats for chimpanzees (*Pan troglodytes*) and gorillas (*Gorilla gorilla*) has declined over time, especially outside protected areas (Strindberg et al. 2018; Heinicke et al. 2019). The related human activities resulted in a decrease in chimpanzee densities as well as behavioural change of primates (Strindberg et al. 2018; Kühl et al. 2019). In addition to human impact, environmental factors such as climate, habitat types and relief play an important role in shaping the occurrence and distribution of chimpanzees (Lehmann et al. 2010; Sesink Clee et al. 2015; Jantz et al. 2016; Abwe et al. 2019; Kalan et al. 2020).

Several Species Distribution Models (SDMs) have been used to map and predict the geographic range of mammalian species (Elith and Leathwick 2009). One of the most commonly used SDMs in this context is the Maximum Entropy (MaxEnt) species distribution model (Elith et al. 2011; Phillips et al. 2006). MaxEnt has been previously used to map and predict habitat suitability for great apes at a continental (Junker et al. 2012) or regional scale (Sesink Clee et al. 2015), and at several study sites across Africa (Table 1).

**Table 1:** Relationship between major predictor variables of suitable great ape habitats and their occurrence in some study sites across Africa

Predictor variable	Great ape occurrence	Country	Study site	References
Road density	Negative	Cameroon	FMUEC	Kehou et al. (2021)
Dense forests	Positive	Cameroon	LNP	Ginath Yuh et al. (2020)
Human impact	Negative	Cameroon	LNP	Ginath Yuh et al. (2020)
Elevation/forest density	Positive	Cameroon	DFR	Tédonzong et al. (2020)
Rainfall/humidity	Positive	Rwanda	NNP	Tuyishimire et al. (2020)
NDVI/elevation	Positive	Guinea	GNL	Fitzgerald et al. (2018)
Elevation/slope	Positive	Nigeria	AMOFL	Onojeghuo et al. (2015)
Elevation/slope	Positive	Cameroon	MH	Etiendem et al. (2013)
Distance to villages	Negative	Cameroon	MH	Etiendem et al. (2013)
Altitude	Negative	Cameroon	MCNP	Mwambo et al. (2010)

*FMUEC* Forest Management Units of Eastern Cameroon, *LNP* Lobeke National Park, *DFR* Dja Faunal Reserve, *NNP* Nyungwe National Park, *GNL* Greater Nimba Landscape, *AMOFL* Afi-Mbe-Okwangwo Forest Landscape, *MH* Mawambi Hills, *MCNP* Mount Cameroon National Park

Despite these previous studies, information is still missing for some protected areas across the distribution range of the Nigeria–Cameroon chimpanzee (Morgan et al. 2011). Junker et al. (2012) reported that the availability of suitable habitat for the Nigeria–Cameroon chimpanzee decreased slightly between the 1990s and 2000s. Sesink Clee et al. (2015) conducted an additional assessment, and their results predicted that suitable habitat for this subspecies in the ecotone region of Cameroon would decline drastically by 2080, while habitat availability in the rainforest region in North-West Cameroon is predicted to remain stable. Onojeghuo et al. (2015) reported that suitable habitats of the Nigeria–Cameroon chimpanzee are facing severe threats from deforestation and forest fragmentation in the Afi Mountain Wildlife Sanctuary, Afi River Forest Reserve, Mbe Mountains and Cross River National Park in the northern part of Cross River State in Nigeria. However, little is known about the actual habitat suitability and availability for the Nigeria–Cameroon chimpanzee, particularly for chimpanzee populations inhabiting forest reserves in the North-West region of Cameroon.

In the North-West region of Cameroon, the Kom-Wum Forest Reserve (KWFR) is a priority conservation site for the Nigeria–Cameroon chimpanzee and in terms of primate diversity in general (Morgan et al. 2011; Doumbé 2015; Chuo et al. 2017; Fotang et al. 2021a). This chimpanzee subspecies is highly threatened by habitat loss and poaching, with approximately 6000 individuals remaining in the wild (Morgan et al. 2011). Previous surveys at this site focused on estimating chimpanzee abundance and habitat preference using the line transect method (Fotang et al. 2021a). Habitat preferences were determined using linear regression techniques (e.g. generalised linear mixed model and multiple linear regression) without informing about any spatial arrangement (Fotang et al. 2021a, b). Modelling suitable habitat for the Nigeria–Cameroon chimpanzee in this reserve using spatial models such as MaxEnt can improve our understanding of their habitat requirement and threats affecting their survival.

So far, data on the availability of suitable habitat for chimpanzees in and around the reserve has yet to be measured. Considering the limited information on suitable habitat for chimpanzees in this reserve, it is important to map and prioritise potential chimpanzee habitat to develop site-specific conservation plans for long-term monitoring of chimpanzee populations. This study, therefore, aims to better understand the habitat requirements of chimpanzees and specifically to (1) map and predict suitable chimpanzee habitat, (2) evaluate the contribution of environmental variables to chimpanzee habitat suitability and (3) determine the probability of chimpanzee occurrence with respect to environmental variables in KWFR using MaxEnt.

## Methods

### Study area

**Fig. 1:**
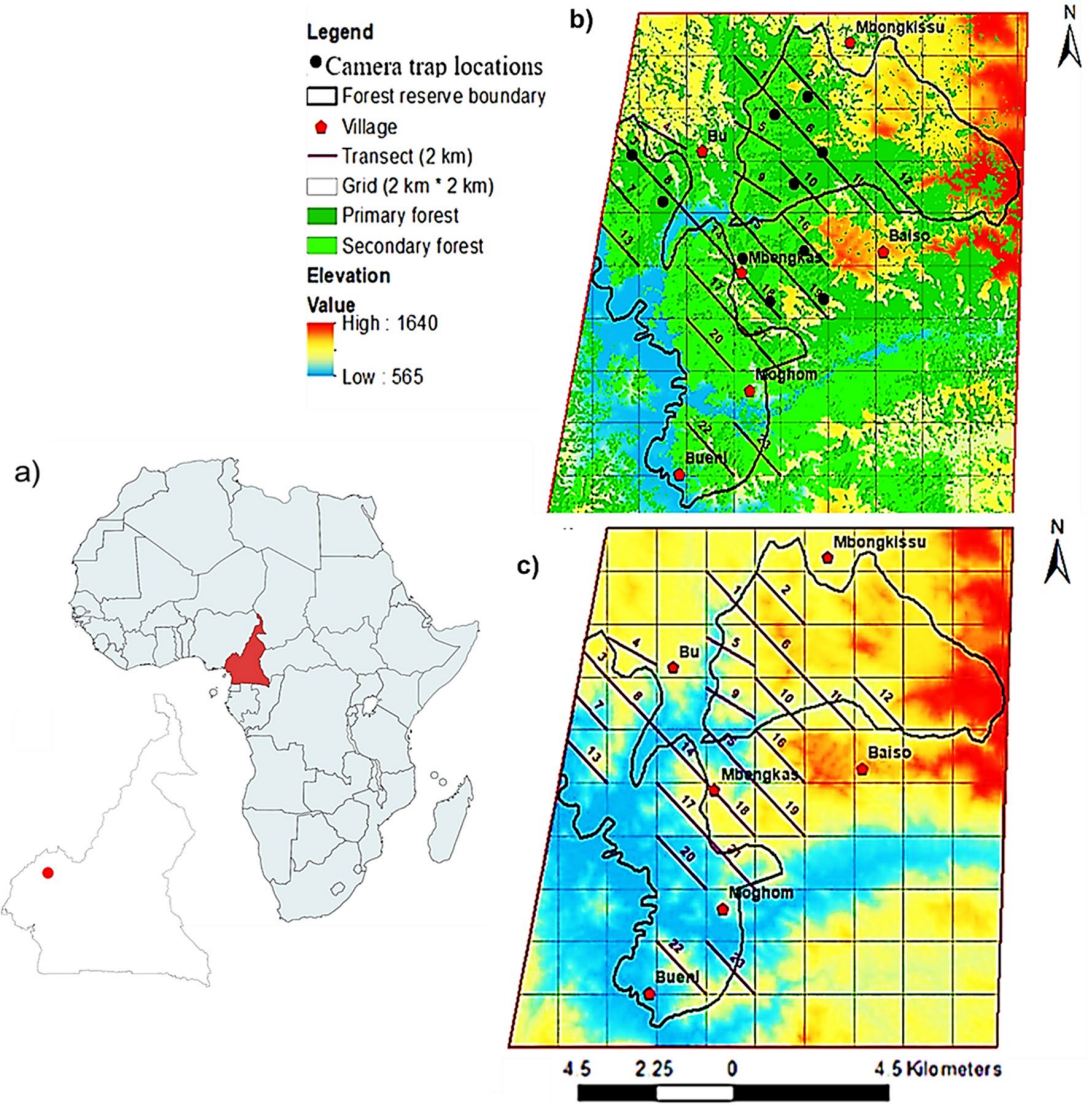
HYPERLINK "sps:id::fig1||locator::gr1||MediaObject::0" Maps of **a** Cameroon and the study location KWFR in North-West Cameroon (red point), **b** map of KWFR including major villages with transects and **c** elevation (Fotang et al. 2021b)

The KWFR stretches across Boyo and Menchum divisions in the North-West region of Cameroon (Fig. 1a; latitude 6°9′39.47″N and longitude 10°13′9.16″E to latitude 6°19′39.42″N and longitude 10°13′3.93″). It has a surface area of about 8029 ha with an elevation of 584–1654 m above sea level (Fig. 1b). The vegetation is dominated by lowland-montane tropical forest species including *Khaya ivorensis*, *Triplochiton scleroxylon*, and *Milicia excelsa* (Morgan et al. 2011). The temperature ranges from 15 °C to 38 °C, with a mean yearly rainfall of about 2400 mm and a humidity of 82% (PNDP 2011). The area has two main seasons, a rainy season (March to October) and a dry season (November to March). The rivers Meteh, Tschuh Akooghe and Mughom flow within the reserve and join the Menchum River that flows at the boundaries of the reserve towards Nigeria (Kah 2015). This reserve harbours seven diurnal and six nocturnal primate species (Doumbé 2015; Chuo et al. 2017; Fotang 2018). About 10–83 chimpanzees live in the reserve (Fotang et al. 2021a). The KWFR is surrounded by the villages Mughom and Bueni Bu, Mbengkas, Baiso and Mbongkissu (Fig. 1b). Hunting and forest degradation are the major threats to forest resources in KWFR (Fotang et al. 2021a, b).

### Survey designs and occurrence data

We produced geo-referenced 2 × 2 km^2^ grids in ArgGis10.6 and superimposed them on a map of the study area (100 km^2^) following a systematic random design (Buckland et al. 2001). We sampled 23 grids (92 km^2^) with spatial line transects (each 2 km in length) linked by 42.09 km recces (Fotang et al. 2021a). In the field, the first author, two experienced forest guides and two community eco-guards repeatedly surveyed recce-transects 16 days every month from May to September 2018 and from November 2019 to March 2020 for signs of chimpanzee activity (chimpanzee occurrence points). We used the recces to access start-of-line transects and increase encounters with chimpanzee signs. We marked the locations of chimpanzee signs using a handheld Global Positioning System (GPS). At nesting sites, we searched for chimpanzee nests within a 50 m radius (White and Edwards 2000). In total, we recorded 653 chimpanzee occurrence points including nesting locations, tool used sites, dung, feeding locations, direct observations, tracks and footprints over a survey effort of 700.9 km for the two survey periods. We re-used chimpanzee sign occurrence points (*N* = 362) recorded during previous line transect surveys between May and September 2018 (Fotang et al. 2021a) and new chimpanzee occurrence points (*N* = 291) recorded during recce and line transect between November 2019 and March 2020 in KWFR. All nests detected during the survey were constructed by chimpanzee, as gorillas are not present in this forest (Doumbé 2015; Chuo et al. 2017; Fotang et al. 2021a). Spatial thinning was done using the thin function in the spThin R package (Aiello-Lammens et al. 2015). The thinning reduced chimpanzee occurrence points to 198 that were used in the final model.

### Environmental variables

**Fig. 2:**
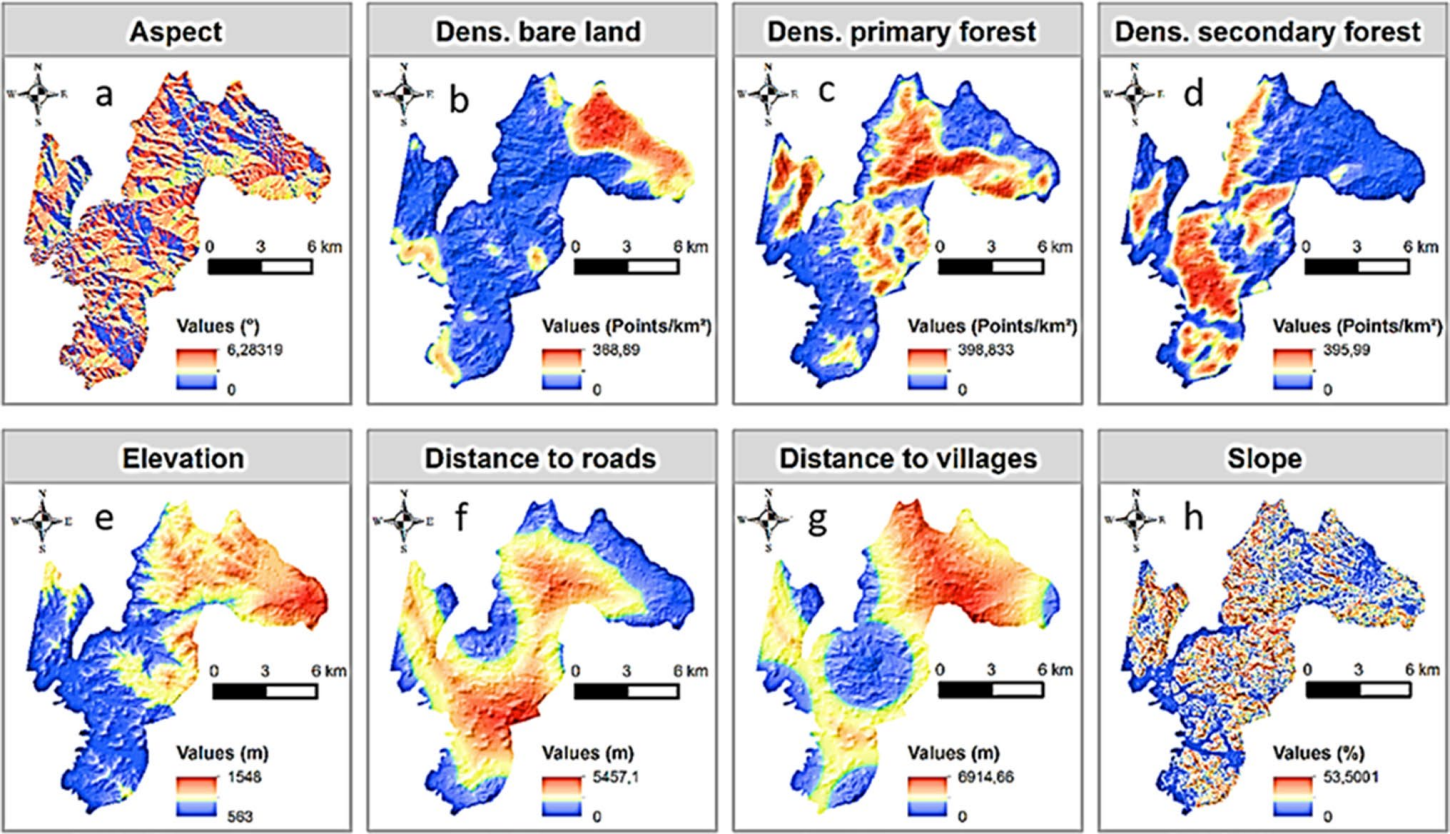
Environmental variable with **a** aspect, **b** density of bare land, **c** density of primary forest, **d** density of secondary forest, **e** elevation, **f** distance to roads, **g** distance to villages and **h** slope

To model the habitat suitability of chimpanzees within the study area, we used nine environmental variables, including aspect, the density of bare land, density of primary forest, density of secondary forest, elevation, distance to roads, distance to villages, distance to rivers, and slope derived from a variety of sources. First, we obtained land cover data (primary forest, secondary forest, bare lands and water bodies) for the study area from a land cover classification map generated by Fotang et al. (2021a). Second, we obtained topographic data by calculating aspect and slope in ArcGIS using elevation data from a Shuttle Radar Topography Mission 30 m resolution Digital Elevation Model (Jarvis 2008). Third, we re-scaled the raster layers of all environmental variables at 50 × 50 m grid cells (pixels). Lastly, we converted the raster layers to points (number of pixels) and used the Kernel Density interpolation method in ArcGIS to calculate the densities of primary forest, secondary forest and bare lands per km^2^ (Fig. 2; Tarjuelo et al. 2017).

Additionally, we used Google Earth images to digitize roads, rivers and villages, and then measured the distance to each feature as Euclidean distance. To check for collinearity between variables, we used the package usdm in R (Naimi et al. 2014). We set a correlation threshold of 0.7 and used the variance inflation factor (VIF) to choose which variable to remove. When the correlation between two variables was greater than a threshold value of 0.7, the variable with the greater VIF was removed, and the correlation matrix was calculated again until the threshold condition was satisfied (Naimi et al. 2014). We only discarded distance to rivers after analyzing for collinearity. Our final models had eight environmental variables, including density of bare land, density of primary forest, density of secondary forest, elevation, distance to roads, distance to villages, aspect and slope.

### Species distribution model

We employed the Maximum Entropy Distribution Model (MaxEnt) version 3.1.4 to predict suitable chimpanzee habitat in relation to eight environmental variables in the study area (Phillips et al. 2006). MaxEnt has many advantages. First, it uses only presence data of a species and produces accurate prediction even with an incomplete dataset and small sample size (Phillips et al. 2006). Second, it uses environmental data from the whole study area rather than only from parts of the area (Phillips and Elith 2013). Third, Maxent uses presence data plus background data (pseudo-absence data) from the study region because true absences are very difficult to obtain, especially for mobile species, and require higher levels of sampling effort to ensure their reliability compared with presence data (Mackenzie and Royle 2005). Fourth, MaxEnt can also be integrated inside other presence-only species distribution models such as Wallace (Kass et al. 2018). Lastly, MaxEnt employs regularization to prevent overfitting that is better than variable-selection methods often used for regression-based models such as generalised linear models (Phillips and Dudík 2008).

We used 198 chimpanzee occurrence points for modelling (178 points for training and 20 for testing). We added 1000 generated background points to the 198 training points, resulting in 1198 points in the final model. We evaluated the performance of the model using the area under the curve (AUC) of the receiver operating characteristic (ROC) (Yackulic et al. 2013). To quantify the habitat suitability of chimpanzee in the reserve, we classified potential chimpanzee habitat into four habitat suitability index scores: highly suitable (> 0.6–1.0), moderately suitable (> 0.4–0.6), low suitable (> 0.2–0.4) and unsuitable habitat (> 0–0.2), following Yang et al. (2013). We then used a jackknife test to measure each environmental variable percentage contribution to chimpanzee habitat suitability (Phillips et al. 2006). We used a logistic output to measure the probability of chimpanzee occurrence with respect to the eight environmental variables (Phillips and Dudík 2008).

## Results

### Suitable habitat area

**Fig. 3:**
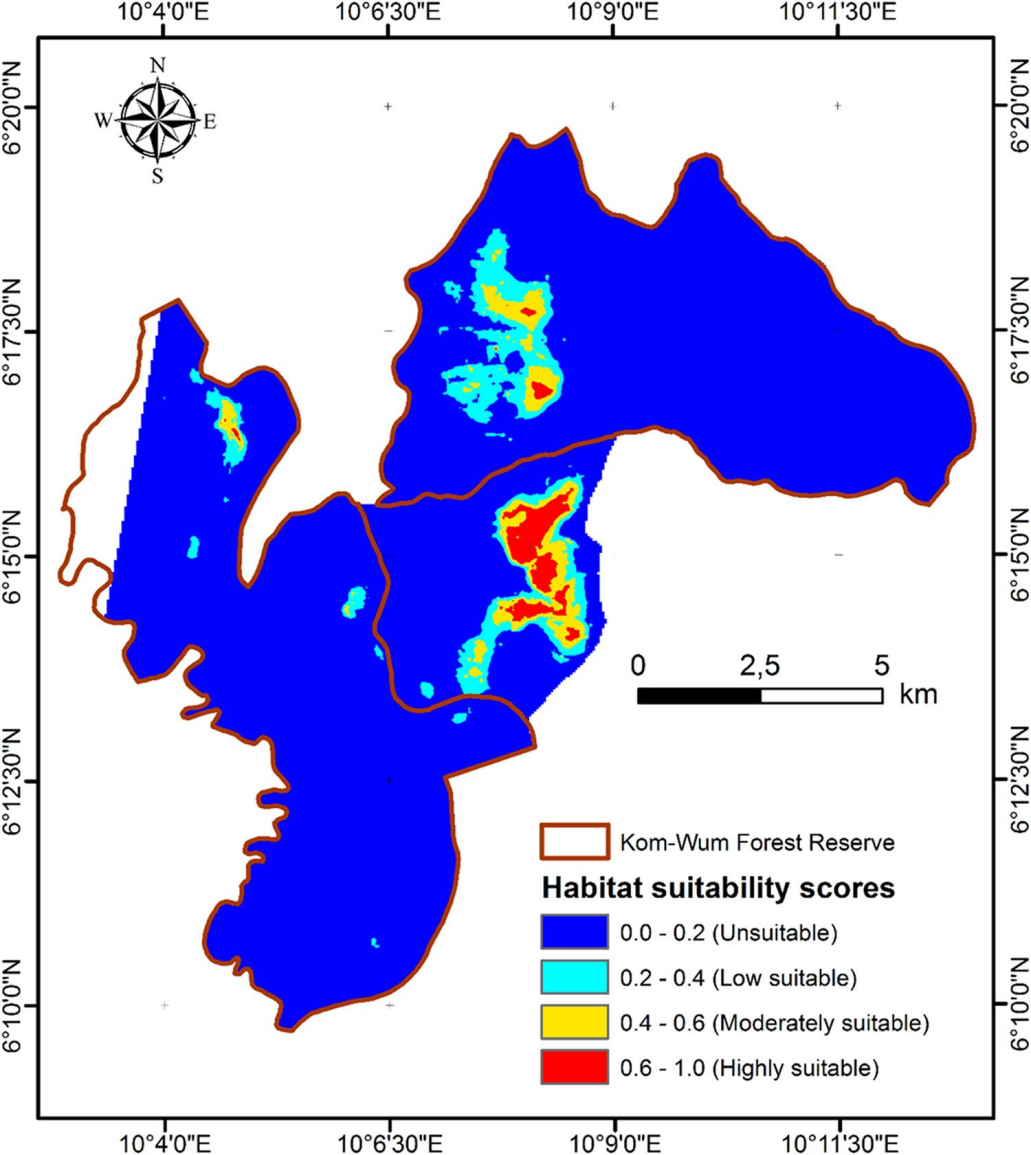
Predicted habitat suitability map for chimpanzee in KWFR

The MaxEnt model fit was very good, with an AUC value of 0.958 (SD ± 0.009). We found that 91% of the study area is unsuitable for chimpanzees, 5% is of low suitability, 3% is moderately suitable and 1% is highly suitable (Fig. 3). In total, 65% of suitable chimpanzee habitat occurred outside the reserve boundary (Fig. 3).

### Per cent contribution of each environmental variable to the MaxEnt model

**Fig. 4:**
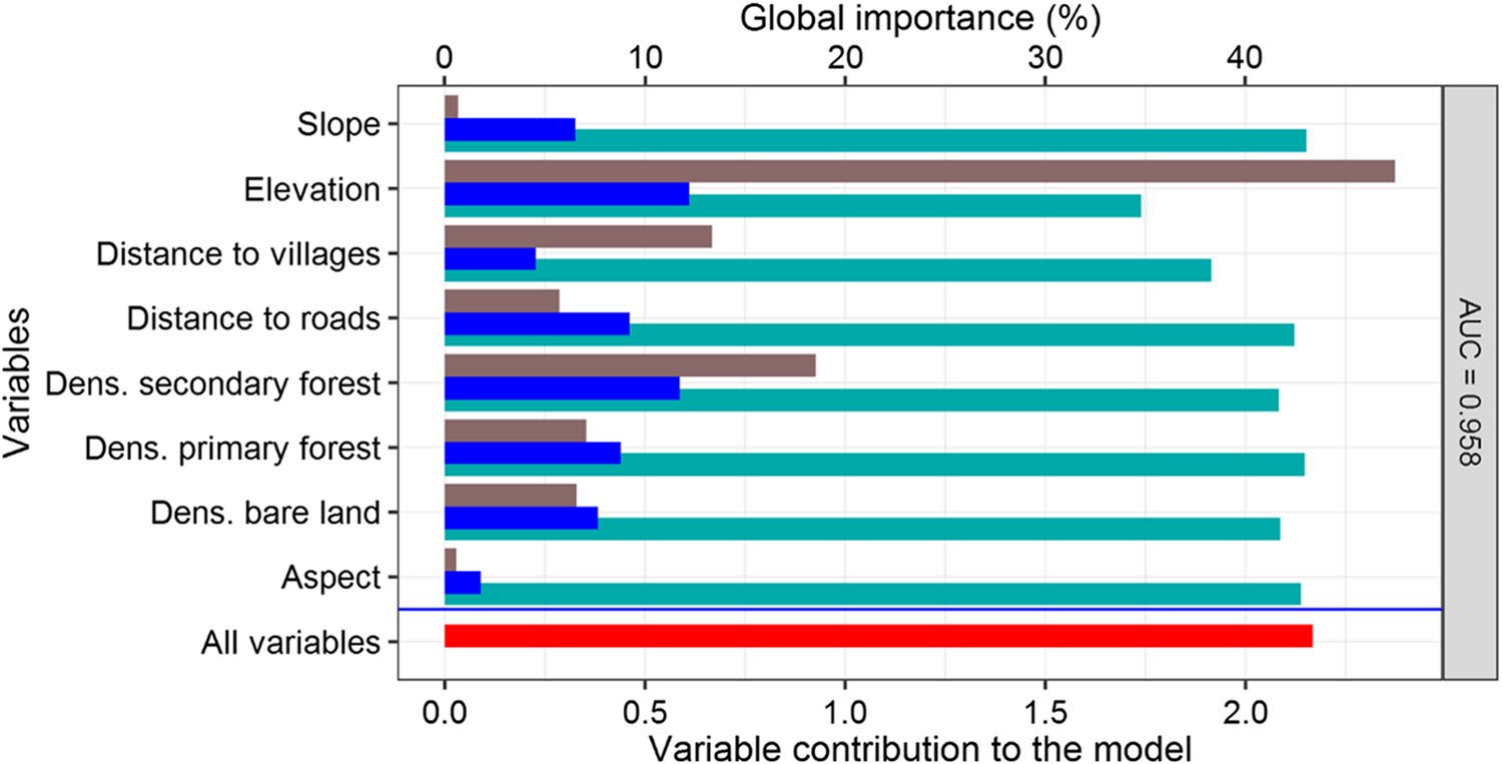
Jackknife regularised training gain and variable contribution to the MaxEnt Model. Blue columns show the model gain when variables are considered alone. Dark-green bars show the training gain without variable. Red bars show the training gain when all variables are used in the model. Brown columns show the global importance

Elevation (42.9%), secondary forest (18.6%), the distance to villages (14.5%) and primary forest (11%) were the highest contributors to chimpanzee habitat suitability in the reserve, and the remaining five environmental variables only contributed 12.6%. If variables are considered alone, the jackknife test supported elevation and density of secondary forests as the most significant contributors to chimpanzee habitat suitability (Fig. 4). The overall contribution of the variables was reduced by 19.8% if elevation was removed, by 11.7% if distance to villages was removed and by 3.9% if density of secondary forests was removed (Fig. 4).

### Variable response curves

**Fig. 5:**
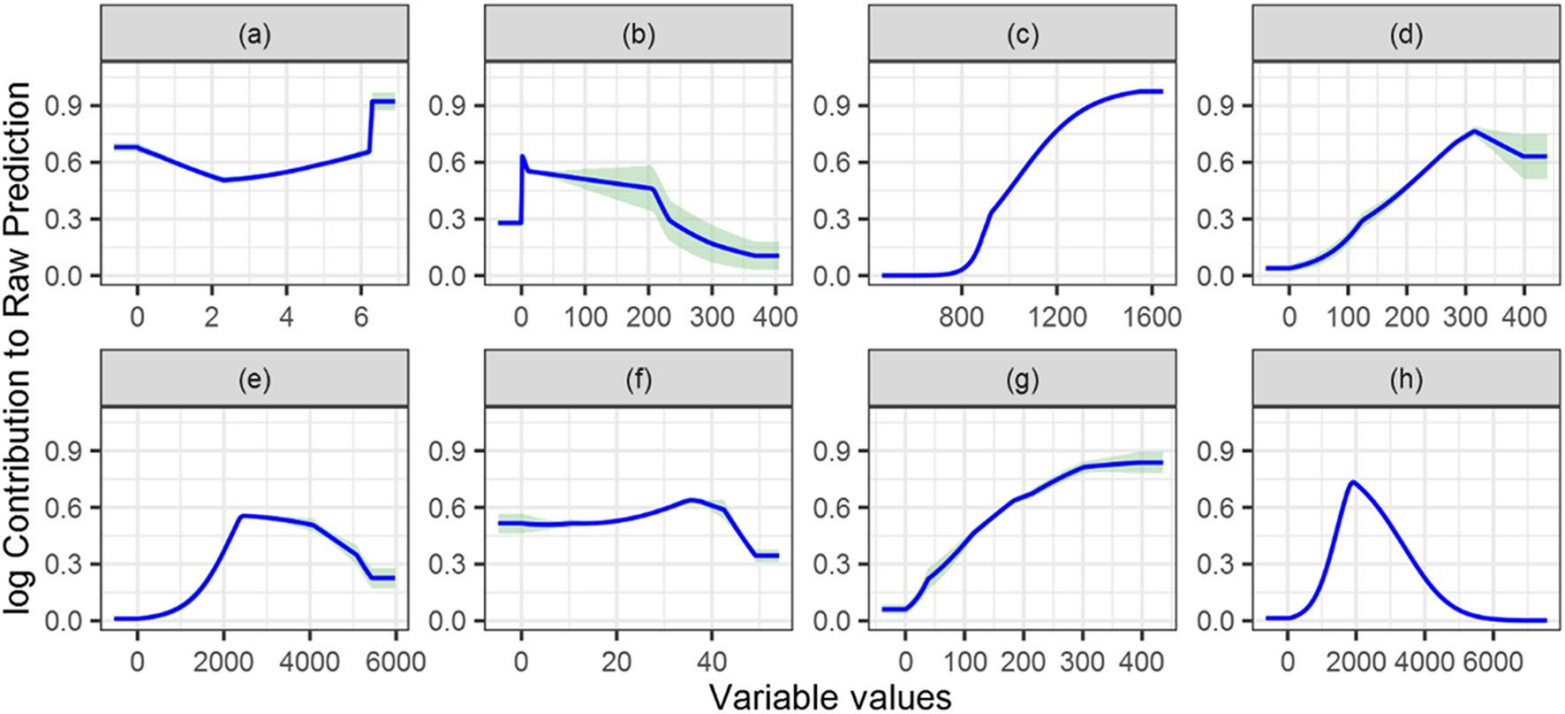
Response curves of chimpanzees to different environment variables with **a** aspect, **b** density of bare land, **c** elevation, **d** density of primary forest, **e** distance to roads, **f** slope, **g** density of secondary forest and **h** distance to villages

The probability of chimpanzee occurrence increased with elevation, slope, and primary and secondary forest density (Fig. 5c, f, d, g). In contrast, chimpanzee occurrence decreased closer to villages, roads and bare land (Fig. 5h, e, b). The probability of chimpanzee occurrence was higher for elevation above 1200 m than 800–1200 m (Fig. 5c). As for the slope, the probability was only slightly higher for the 20–40° than for the less than 20° section of the curves and dropped significantly after 40° (Fig. 5f). Chimpanzees were less likely to be found in areas closer than 2000 m to roads and villages (Fig. 5h, e). The probability of occurrence started to increase in areas with a density of less than 100 points (number of pixels) per square kilometre in primary and secondary forests and peaked at 300 points (Fig. 5d, g).

## Discussion

Suitable chimpanzee habitat is degraded in the study area and this population could go extinct unless immediate action is taken. Elevation, secondary forest density, distance to villages and primary forest density were the most important predictors for habitat suitability in the study area. These results are alarming, as the proportion of suitable habitat for this rare chimpanzee subspecies in KWFR is one of the lowest compared with values reported at other sites across its distribution range. Although suitable chimpanzee habitat in the North-West region of Cameroon are predicted to remain intact in the next six decades (Sesink Clee et al. 2015), chimpanzee habitats in KWFR are already scarce, and may no longer be suitable in the near future. Our results highlight the urgent need to improve the management of this protected area.

### Suitable habitat area

The area of suitable chimpanzee habitat is small in the study region, and a large proportion of highly suitable chimpanzee habitat occurs outside the reserve. Although the natural vegetation cover is much larger, chimpanzees are confined to less than 10% of the area (Fig. 3) because of illegal gun hunting, trapping, harvesting of timber and non-timber forest product, extensive cattle grazing, bushfires, cattle rearing and habitat destruction for the creation of new settlements in other parts of the reserve (Morgan et al. 2011; Kah 2015; Fotang 2020; Fotang et al. 2021a). As a result, chimpanzees of KWFR occupy core habitat even outside the protected area, which may expose them to further hunting and increased risk of local extinction (Crooks et al. 2017; Heinicke et al. 2019). The preference for habitat outside KWFR may result from low human pressure, high availability of chimpanzee preferred fruits and nesting sites in these areas (Basabose and Yamagiwa 2002; Abwe et al. 2019).

The proportion of suitable chimpanzee habitat in KWFR (9%) is among the lowest compared with those reported at other sites across this subspecies distributional range, including 1.9% in Afi River Forest Reserve, 14.3% in Mbe Mountains, 29.4% in Afi Mountain Wildlife Sanctuary and 54.4% in Cross River National Park in Nigeria (Onojeghuo et al. 2015). Similarly, suitable chimpanzee habitat in KWFR is lower than 61.0% reported in Forest Management Units of Mindourou, Lomié and Messok towns in the Eastern region of Cameroon (Kehou et al. 2021), 67.4% in Mount Cameroon National Park (Mwambo 2010) and 71.0% in Lobéké National Park in South‐East Cameroon (Ginath Yuh et al. 2020).

### Environmental variables in suitable areas

Elevation showed the highest contribution in predicting suitable chimpanzee habitat as chimpanzee occurrence increased with elevation. The increase in the probability of chimpanzee occurrence between 800 and 1200 m is best explained by the steep slopes. The preference for high elevation and steep terrain in this study could be a way of escaping human activities in other areas of the reserve (Fotang et al. 2021b). High elevation areas are not suitable for farming and are very difficult to access by illegal timber exploiters and poachers; therefore, these areas are relatively safe for chimpanzees (Fotang et al. 2021a). The lowland areas (< 800 m) are often cultivated with rice and maize and suffer from logging, fishing and hunting along the banks of rivers Menchum, Tschuh Akooghe and Mughom (Kah 2015; Chuo et al. 2017; Fotang et al. 2021b). Our results support previous work that elevation is the best predictor of suitable chimpanzee habitat (Jantz et al. 2016). Elevation was the best predictor of chimpanzee habitat suitability in the northern periphery of Dja Faunal Reserve in Cameroon (Tédonzong et al. 2020). At Seringbara, Nimba mountains, chimpanzees occurred in areas between 800 and 1000 m for feeding and areas above 1000 m for nesting (Koops et al. 2012). Further surveys at Seringbaran revealed that the suitability of chimpanzee habitat increased above 700 m due to the absence of crop fields (Fitzgerald et al. 2018). At Tofala Hill Wildlife Sanctuary in Cameroon, the selection of nesting sites at higher altitudes (800–1000 m) by chimpanzees was linked to the avoidance of high encounter rates with agricultural and logging activities at lower altitudes (Njukang et al. 2019). Etiendem et al. (2013) indicated that elevation contributed most to suitable habitat for the cross river gorillas at Mawambi Hills, South-West Cameroon.

Secondary forest density showed the second highest contribution to suitable chimpanzee habitat, and the probability of chimpanzee occurrence increased with the density of secondary forest. In contrast, Fotang et al. (2021a) showed that secondary forests cover has a significant negative effect on the occurrence of chimpanzee signs. The positive evaluation of secondary forest in this paper suggests that secondary forest vegetation itself is not necessarily negative for chimpanzees, and the negative evaluation by Fotang et al. (2021a) is due to the high level of human activity in secondary forests. This means that chimpanzees do not seem to avoid secondary forest because they are unsuitable but are ‘unwillingly’ compelled to do so because of high human disturbance in secondary forest. Therefore, the suitable habitat area defined by SDMs in our analysis (Fig. 3), is a habitat in which the probability of chimpanzee occurrence is high and not necessarily one that is truly favourable for their survival and reproduction. While it is important to conserve the areas designated as suitable chimpanzee habitats in this paper in the short term (Fig. 3), it is also crucial to reduce the impact of human activities in other areas (secondary forest in particular) to secure chimpanzee populations in reserve in the long term (100 to several hundred years). Our results suggest that secondary forests are suitable chimpanzee habitats and should be considered for effective conservation planning in the area.

Distance to villages was the third most important contributor to suitable chimpanzee habitat, followed by distance to roads and density of bare lands. Our results revealed that chimpanzees avoided villages, roads and bare lands. The low occurrence of chimpanzees close to villages and roads (< 2000 m) could be explained by the conversion of dense forest vegetation into maize fields and bare lands for the establishment of new settlements. Similarly, at Cantanhez National Park Guinea-Bissau, chimpanzees forage frequently in forested areas far away from villages (Bersacola et al. 2021). In non-protected areas of Tanzania, chimpanzee densities were low close to settlements due to the destruction of chimpanzee habitat through cultivation (Ogawa et al. 2006, 2013). At Mawambi Hills, South-West Cameroon, suitable habitat for great apes was low close to villages (Etiendem et al. 2013). Chimpanzees avoided roads in KWFR because roads lead to rice fields and are usually surrounded by maize and bean fields. Pedestrians and motorcycles frequently use these roads to transport farm products to neighbouring villages. Our results support data from south-western Sierra Leone where chimpanzees avoid roads and use areas that are not cultivated by farmers (Garriga et al. 2019). In contrast to our findings, chimpanzees did not avoid roads in Cantanhez National Park Guinea-Bissau because these roads are located in the centre of their home range (Bersacola et al. 2021).

Primary forest vegetation was the fourth most important contributor to suitable chimpanzee habitat and slopes contributed little to overall suitability of chimpanzee habitat in the study area. We observed a positive correlation between chimpanzee occurrence and the density of primary forest and steep slopes. The preference for primary forest by Kom-Wum chimpanzees could be explained by the availability of their preferred nesting and feeding trees, while the selection of steep slope could be due to low encounter rate of human activities in these areas (Fotang et al. 2021a). In the northeastern part of the Nimba Mountains in Côte d'Ivoire and Guinea, the probability of finding chimpanzee nests increased in primary forests, especially on steep slopes (Granier et al. 2014). Chimpanzee nesting habitat preference in Lagoas de Cufada Natural Park (LCNP) was associated with dense canopy forest (Sousa et al. 2014). At the Greater Mahale Ecosystem in Tanzania, nesting on steep slopes was identified as a predator avoidance strategy (Chitayat et al. 2021).

### Limitation of the study

We did not include climatic variables in our model. We, therefore, recommend that future models should include additional environmental factors that may impact the habitat suitability of chimpanzees, such as temperatures and rainfall (Lehmann et al. 2010).

## Conclusions

The majority of areas in KWFR are unsuitable habitats for chimpanzees, and a high proportion of highly suitable habitat is located outside the protected area. Optimum chimpanzee habitat was found at high elevations (800–1200 m) more than 2000 m further from villages and roads, and with no bare lands. In response, the likelihood of finding chimpanzees increased with elevation, secondary forest density and primary forests density, and decreased with the density of bare land, distance to villages and roads. While elevation remains the strongest predictor of suitable chimpanzee habitat in the area, dense secondary forests are crucial for the expansion of the chimpanzee population in the area if human activities are reduced. Our findings suggest that dense secondary forests and primary forests are suitable habitats for chimpanzees in the reserve and should be included in future conservation plans. Protected area managers have to focus on reducing human activities in secondary forests such as illegal gun hunting, trapping, harvesting of timber and non-timber forest, illegal logging, forest conversion to farmland and settlements to secure the remaining suitable habitats and the chimpanzee population in the reserve. In addition, the reserve boundary should be extended to include suitable chimpanzee habitat outside the reserve in future management plans.

## Data Availability

The datasets generated and analysed for the current study are available from the corresponding author on reasonable request.
